# Collaboration Between Acute Care Hospitals and Nursing Homes for Dysphagia Management: A Comparative Study of Patients With and Without Pneumonia-Related Hospitalization

**DOI:** 10.7759/cureus.81400

**Published:** 2025-03-29

**Authors:** Takafumi Yamano, Shoichi Kimura, Fumitaka Omori, Kaori Wada, Miho Tanaka, Takashi Tsutsumi

**Affiliations:** 1 Section of Otorhinolaryngology, Department of Medicine, Fukuoka Dental College, Fukuoka, JPN; 2 Department of Otorhinolaryngology, Fukuoka Dental College Hospital, Fukuoka, JPN; 3 Department of Nursing, Fukuoka Dental College Hospital, Fukuoka, JPN; 4 Section of the Center for Visiting Dental Service, Department of General Dentistry, Fukuoka Dental College, Fukuoka, JPN

**Keywords:** dysphagia, fiberoptic endoscopic evaluation of swallowing, nursing homes, recurrent aspiration pneumonia, videofluoroscopic swallowing studies

## Abstract

Objective

Although acute care hospitals address pneumonia through antibiotic therapy and provide in-hospital swallowing rehabilitation conducted by speech-language pathologists, post-discharge follow-up is often insufficient. In particular, limited attention is given to monitoring the recurrence of pneumonia and the status of oral intake once residents return to their nursing homes. The aim of this study was to determine the benefits of collaboration between acute care hospitals and nursing homes for dysphagia and whether swallowing tests are a predictor of the development of pneumonia.

Methods

We included 48 residents (17 males and 31 females) from affiliated nursing homes who underwent swallowing function assessments at our hospital between April 2018 and September 2024. The mean age of the participants was 88 (72-107) years. The participants were divided into two groups based on the presence or absence of hospitalization for pneumonia. Swallowing function was assessed using videofluoroscopic swallowing studies (VFSS) and fiberoptic endoscopic evaluation of swallowing (FEES). The Mann-Whitney U test was used to compare the two groups. Statistical analyses were performed using SPSS version 29.0 (IBM Corp., Armonk, NY).

Results

The primary presenting complaint leading to consultation was coughing during meals, observed in eight (25.8%) non-hospitalized and nine (52.9%) hospitalized patients. No statistically significant differences were observed in oral hygiene status, as measured by the Oral Health Assessment Tool (OHAT) score, between the two groups. However, the Hyodo score obtained from FEES was significantly higher in the hospitalized group compared to the non-hospitalized group (P = 0.004). Similarly, the score on the Penetration-Aspiration Scale (PAS) from VFSS was significantly higher in the hospitalized group (P = 0.002).

Conclusion

Interinstitutional collaboration between acute care hospitals and nursing homes in the assessment of swallowing function provides significant benefits for both settings. In addition, in elderly care facilities with well-established oral care practices, optimized mealtime environments, and standardized feeding assistance protocols, instrumental swallowing function assessments demonstrate significant prognostic utility for predicting pneumonia onset.

## Introduction

Fukuoka Dental College Medical and Dental General Hospital has implemented a standardized protocol for the management of dysphagia. This protocol mandates that all patients undergo videofluoroscopic swallowing studies (VFSS) and fiberoptic endoscopic evaluation of swallowing (FEES) to inform the development of individualized treatment plans based on comprehensive examination findings [1]. A distinguishing feature of our hospital is the high volume of swallowing function assessment requests received from affiliated nursing homes. In these facilities, accurate diagnosis and effective management of dysphagia are essential for the prevention of pneumonia and choking among residents [2]. Although acute care hospitals address pneumonia through antibiotic therapy and provide in-hospital swallowing rehabilitation conducted by speech-language pathologists, post-discharge follow-up is often insufficient. In particular, limited attention is given to monitoring the recurrence of pneumonia and the status of oral intake once residents return to their nursing homes. We evaluated the potential benefits of collaboration between acute care hospitals and nursing homes in the assessment and management of swallowing function. Furthermore, we investigated the predictive utility of swallowing function tests as indicators of pneumonia risk in nursing home residents.

## Materials and methods

Collaboration between the hospital and nursing homes

**Figure 1:**
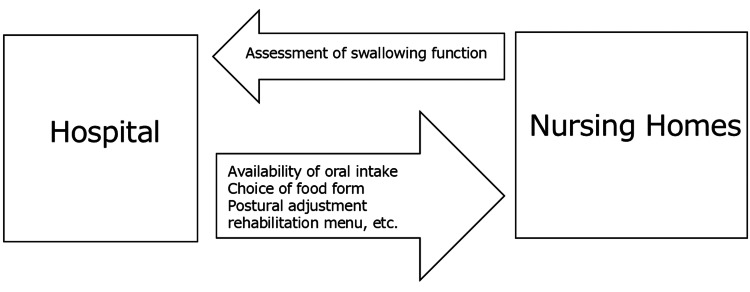
Collaborative framework between the hospital and nursing homes Swallowing assessments are carried out in the hospital, and this information is passed on to nursing homes.

Figure 1 presents the collaborative framework between the hospital and nursing homes.

Upon the identification of concerns regarding a resident's oral intake, either the designated nursing home dentist responsible for domiciliary visits or a facility-based dental hygienist initiates contact with the hospital to request a comprehensive swallowing function assessment. Upon referral, comprehensive swallowing evaluations are conducted at the hospital. Based on the assessment's findings, individualized recommendations are provided regarding the safety of oral intake, appropriate food textures, optimal posture during meals, and the need for targeted rehabilitation programs. Considering the nursing home's affiliation with a dental college, a robust oral care system was established. Participants receive routine oral hygiene care, supervised by dental hygienists, following each meal, and undergo weekly dental examinations conducted by a dentist. Furthermore, interdisciplinary support is provided through regular visits by speech-language pathologists and nurses specializing in dysphagia. These professionals conduct meal rounds, assess residents' eating behaviors, and offer guidance on modifications to food texture and posture to enhance swallowing safety. In addition, they play a crucial role in identifying residents who may require further swallowing function testing.

Subjects

We included 48 residents (17 males and 31 females) from affiliated nursing homes who underwent swallowing function assessments at our hospital between April 2018 and September 2024. The mean age of the participants was 88 (72-107) years. Patients with a history of treatment for head and neck cancer or esophageal cancer were excluded. All participants exhibited dependence in all activities of daily living. Although they demonstrated the capacity to comprehend and execute simple commands, their ability to express intentions was limited. They are also unable to excrete, bathe, or change clothes alone without assistance. Complications related to swallowing function included eight cases of cerebrovascular disease, one case of multiple system atrophy, and one case of progressive epiphysis. Following the swallowing assessments, participants were categorized as those who did not require hospitalization for pneumonia (n = 31) and those who experienced hospitalization for pneumonia (n = 17). Subsequently, comparative analyses were conducted comparing these groups.

A diagnosis of pneumonia was made in cases fulfilling the following criteria: chest X-ray or chest computed tomography (CT) showing an alveolar infiltration shadow, with a fever of 37.5°C or higher and an abnormally high C-reactive protein level, a peripheral white blood cell count of more than 9,000/μL, and/or the presence of any two or more airway symptoms, such as sputum.

Evaluation methods

Oral Health Assessment

Oral health status was evaluated using the Oral Health Assessment Tool (OHAT) [3]. Assessments were performed by either dentists or dental hygienists with expertise in geriatric oral care. The OHAT comprises eight domains: lips, tongue, gingival mucosa, saliva, remaining teeth, dentures, oral cleanliness, and tooth pain. Each item was rated on a three-point scale as follows: healthy, changes, or unhealthy.

FEES

Swallowing function was assessed using the Hyodo scoring system during FEES examinations conducted by two otolaryngologists specializing in dysphagia [4]. During outpatient otolaryngology consultations, patients were positioned in a seated or semi-seated posture. A flexible endoscope was inserted transnasally through the wider nasal cavity. Next, patients were instructed to swallow 3 mL of dyed water. Four parameters were evaluated using a four-point scale: accumulation of saliva in the valleculae and pyriform sinuses, the elicitation of cough and glottic closure reflexes, the initiation of the pharyngeal swallow reflex, and the efficacy of pharyngeal clearance following the swallowing of dyed water.

VFSS

Swallowing function was evaluated using the Penetration-Aspiration Scale (PAS) during VFSS examinations performed by two otolaryngologists specializing in dysphagia [5]. Assessments were conducted in a fluoroscopy suite, with patients seated or positioned in a semi-seated posture. Lateral view fluoroscopic images were obtained to visualize the swallowing process. During the examination, patients were instructed to swallow 10 mL of liquid contrast medium. Swallowing safety was evaluated using the following eight-point PAS: no laryngeal penetration; laryngeal penetration that is expelled without entering the vocal cords; laryngeal penetration that does not reach the vocal cords and is not expelled; laryngeal penetration that reaches the vocal cords but is expelled; laryngeal penetration that reaches the vocal cords and is not expelled; aspiration below the vocal cords that is expelled from the airway; aspiration below the vocal cords accompanied by coughing but not expelled; and aspiration below the vocal cords with no attempt to expel.

Statistical analysis

The Mann-Whitney U test was used to compare the two groups. Statistical analyses were performed using SPSS version 29.0 (IBM Corp., Armonk, NY). The Fukuoka Gakuen Research Ethics Committee approved the study protocol (approval number: 314).

## Results

Reasons for consultation

Table 1 presents the distribution of consultation reasons across the two study groups. Choking during meals was the most frequently cited concern in both the non-hospitalized pneumonia group (n = 8, 25.8%) and the hospitalized pneumonia group (n = 9, 52.9%). The second most common indication for consultation was the determination of optimal food texture, with a higher prevalence in the non-hospitalized pneumonia group (n = 7, 22.6%) compared to the hospitalized pneumonia group (n = 3, 17.6%). The assessment of oral intake feasibility was reported in three cases in each group (9.7% and 17.6%). Coughing was observed in four cases (12.9%) in the non-hospitalized pneumonia group and one case (5.9%) in the hospitalized pneumonia group. Concerns regarding the presence or absence of aspiration prompted consultation in one case from each group (3.2% and 5.9%). Additional reasons for consultation included further refinement of food texture, reassessment of oral intake feasibility, management of persistent coughing, and evaluation of aspiration status. Notably, prolonged mealtime (two cases, 6.5%), decreased appetite (two cases, 6.5%), and discomfort in the pharyngeal or laryngeal region (four cases, 12.9%) were observed exclusively in the non-hospitalized pneumonia group.

**Table 1:** Distribution of consultation reasons across the two study groups

Variables	Non-hospitalized pneumonia group (n = 31)	Hospitalized pneumonia group (n = 17)
Choking during meals	8 (25.8%)	9 (52.9%)
Determination of optimal food texture	7 (22.6%)	3 (17.6%)
Assessment of oral intake	3 (9.7%)	3 (17.6%)
Coughing	4 (12.9%)	1 (5.9%)
Evaluation of aspiration status	1 (3.2%)	1 (5.9%)
Prolonged mealtime	2 (6.5%)	0 (0%)
Decreased appetite	2 (6.5%)	0 (0%)
Pharyngeal or laryngeal region	4 (12.9%)	0 (0%)

OHAT

**Figure 2:**
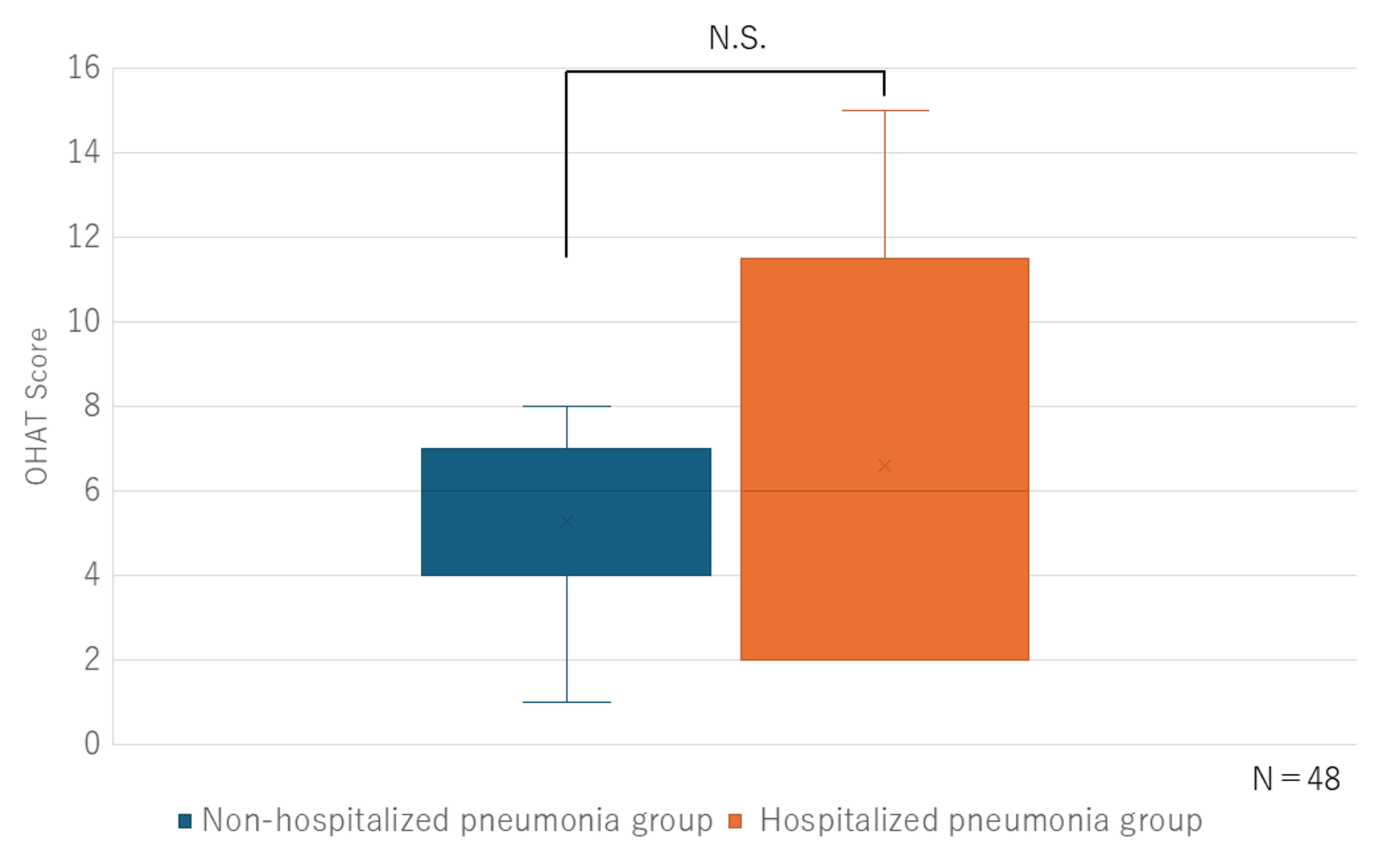
Comparative analysis of OHAT scores conducted between the non-hospitalized and hospitalized pneumonia groups OHAT: Oral Health Assessment Tool

A comparative analysis of OHAT scores was conducted between the non-hospitalized and hospitalized pneumonia groups. Notably, higher OHAT scores indicate poorer oral health conditions. No statistically significant differences in OHAT scores were observed between the groups (n = 48) (Figure 2). This was also the case in the examination of eight domains by subdivision.

FEES

**Figure 3:**
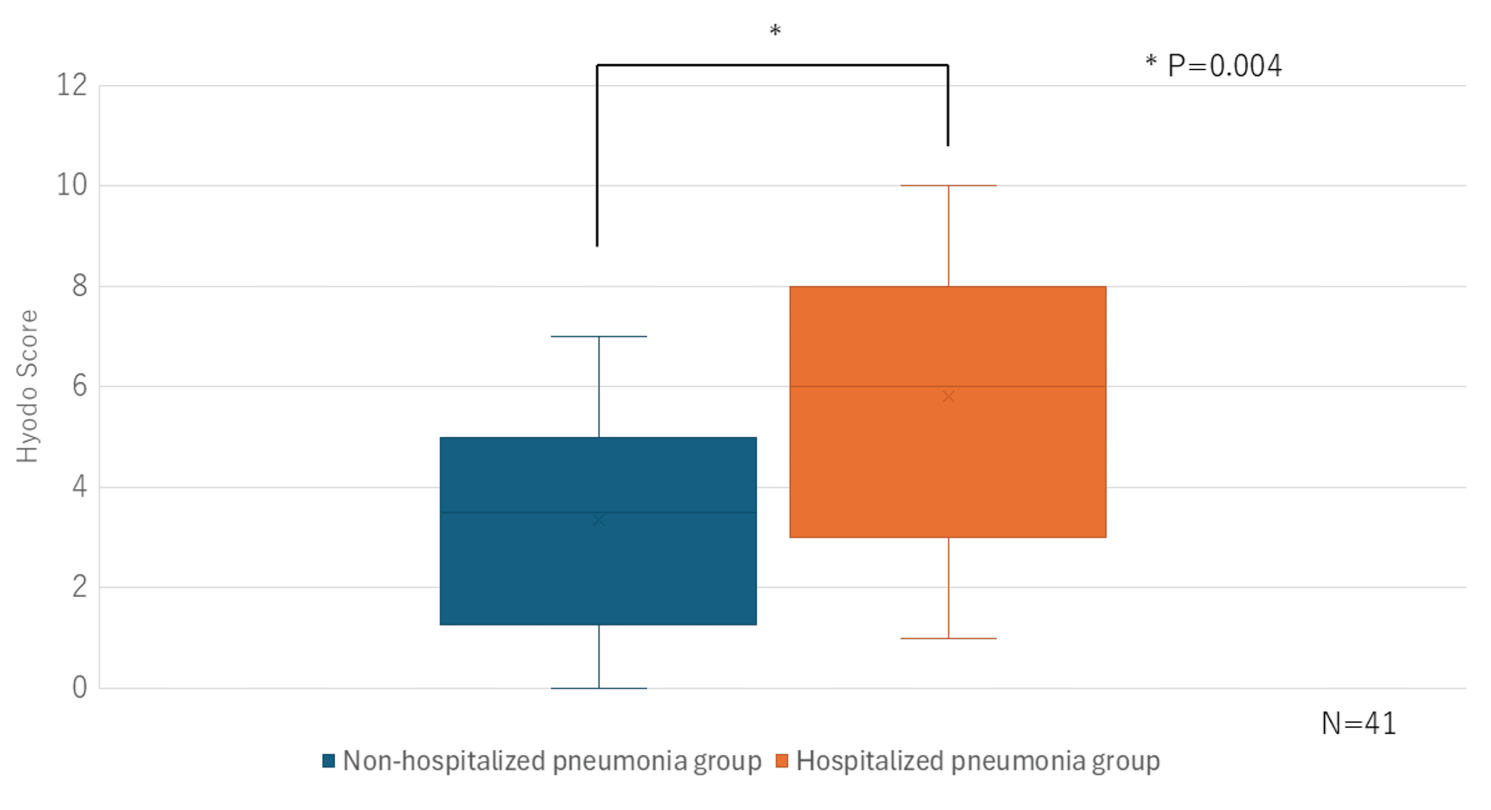
Comparative analysis of Hyodo scores conducted between the non-hospitalized and hospitalized pneumonia groups FEES: fiberoptic endoscopic evaluation of swallowing

Seven participants were excluded from the FEES analysis due to either refusal to undergo nasoendoscopy or cognitive impairment that precluded adherence to examination instructions. Consequently, the final sample for the FEES analysis was 41 participants. Notably, a higher Hyodo score indicates more severe dysphagia. The hospitalized pneumonia group demonstrated a significantly higher Hyodo score compared to the non-hospitalized pneumonia group (P = 0.004) (Figure 3).

VFSS

**Figure 4:**
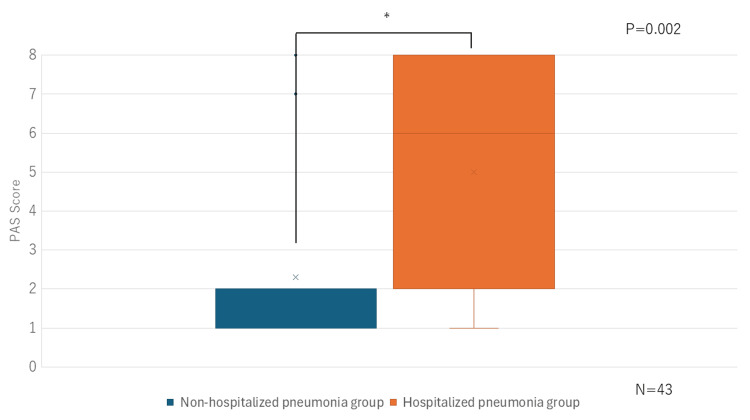
Comparative analysis of PAS scores conducted between the non-hospitalized and hospitalized pneumonia groups VFSS: videofluoroscopic swallowing studies, PAS: Penetration-Aspiration Scale

Five participants were excluded from the VFSS analysis due to cognitive impairment that prevented compliance with the examination, resulting in a final sample size of 43 participants. In the PAS, higher scores reflect a poorer swallowing function. Our findings demonstrated that PAS scores were significantly higher in the hospitalized pneumonia group compared to the non-hospitalized pneumonia group (P = 0.002) (Figure 4).

## Discussion

Significance of collaboration between acute care hospitals and nursing homes

Aspiration pneumonia is a significant contributor to increased mortality among residents of long-term care facilities, emphasizing the importance of early diagnosis and prompt management of dysphagia in these settings [2]. However, professional interventions are often limited due to staffing shortages, particularly the insufficient availability of speech-language pathologists. Consequently, dietary modifications, such as adjustments to food texture, are frequently used as the primary strategy to mitigate the risk of aspiration [6]. Although simple screening methods, such as the modified water swallowing test, have been used to assess swallowing function in elderly care settings, comprehensive evaluations using instrumental assessments such as FEES and VFSS remain uncommon [7]. In addition, significant regional and international disparities exist in the availability of comprehensive swallowing assessments, largely due to limitations in equipment and specialized personnel [8].

We used FEES and VFSS to assess aspiration risk, determine appropriate food textures, and evaluate the feasibility of oral intake in residents presenting with choking during meals. Our findings indicate that these assessments are valuable tools for identifying aspiration risk, guiding the selection of suitable food textures, and determining the safety of oral intake in residents of care facilities who experience meal-related choking. In elderly care settings, this approach contributes to the prevention of aspiration pneumonia and enhances the safety of mealtime support. Furthermore, for hospitals, access to prehospitalization swallowing function data enables the early initiation of swallowing rehabilitation and the timely resumption of oral intake after admission, providing reciprocal benefits for care facilities and hospitals.

Significance of swallowing function tests as predictors of pneumonia onset

Dysphagia is a well-established risk factor for pneumonia in the elderly. However, several studies suggest that dysphagia alone may be insufficient to cause pneumonia in the absence of additional risk factors [9]. For instance, in patients with ischemic stroke, age and neurological severity have been identified as stronger predictors of pneumonia than VFSS [10]. Conversely, other studies have demonstrated that the presence of laryngeal penetration, aspiration, or silent aspiration detected during VFSS increases the risk of pneumonia by approximately 4-, 10-, and 13-fold, respectively [11].

In our study, no significant differences in OHAT scores, which reflect oral health status, were observed between groups. Conversely, significant differences were observed in FEES and VFSS findings between the non-hospitalized and hospitalized pneumonia groups. FEES is a simple and effective method for predicting aspiration and is comparable to VFSS in assessing the oral and pharyngeal phases of swallowing function [12]. A Hyodo score of ≥6 represents a significantly increased aspiration risk [13]. VFSS, which allows comprehensive assessment of the oral, pharyngeal, and esophageal phases, remains the most reliable modality for evaluating swallowing function. Several studies using the PAS have emphasized the association between swallowing dysfunction and pneumonia risk. Ko et al. reported that, among elderly individuals (aged ≥ 65 years), those in the pneumonia group exhibited a higher proportion of male participants and significantly elevated PAS scores compared with the non-pneumonia group [14]. Similarly, Kim et al. demonstrated a significant association between PAS scores ≥ 8 and the onset of pneumonia [15]. Consistent with these findings, our previous study exhibited no correlation between OHAT scores and VFSS findings among elderly care facility residents hospitalized for pneumonia [1]. Our study further supports the notion that oral health status alone is not a decisive factor in the development of aspiration pneumonia in the elderly. Therefore, even among nursing home residents who receive thorough oral care, appropriate meal environment management, feeding assistance, and swallowing function assessments may serve as valuable predictors of pneumonia onset.

Study limitations

Although our study demonstrated the predictive value of swallowing function tests for pneumonia, pneumonia may still manifest in certain individuals despite favorable test findings. This discrepancy may be influenced by factors such as fluctuations in physical condition and alterations in consciousness levels. Furthermore, the result is restricted to facilities with good oral healthcare, and it is possible that this result may not apply to other facilities. These factors were not comprehensively evaluated in our study, emphasizing the need for further studies.

## Conclusions

The assessment of residents' swallowing function in a collaborative setting between acute care hospitals and nursing homes was useful in determining whether residents aspirated during meals and selected food forms and whether oral intake was possible. Swallowing function tests (videoendoscopy (VE) and videofluoroscopy (VF)) may be a predictor of pneumonia only in nursing homes where oral care, eating environment, and assistance methods are thorough.
